# CT-Scan-Assessed Body Composition and Its Association with Tumor Protein Expression in Endometrial Cancer: The Role of Muscle and Adiposity Quantities

**DOI:** 10.3390/cancers16244222

**Published:** 2024-12-18

**Authors:** Cuthbert Mario Mahenge, Rand Talal Akasheh, Ben Kinder, Xuan Viet Nguyen, Faiza Kalam, Ting-Yuan David Cheng

**Affiliations:** 1 Division of Cancer Control and Prevention, Department of Internal Medicine, College of Medicine, The Ohio State University, 3650 Olentangy River Rd., Suite 200, Columbus, OH 43214, USA; mahenge.1@osu.edu (C.M.M.); rand.akasheh@osumc.edu (R.T.A.); faiza.kalam@osumc.edu (F.K.); 2 Department of Radiology, College of Medicine, The Ohio State University, 395 W 12th Ave., Suite 486, Columbus, OH 43210, USA; xuan.nguyen@osumc.edu

**Keywords:** computed tomography, adipose tissues, skeletal muscles, body composition, tumor protein expression, endometrial cancer, signaling pathway

## Abstract

**Background**: Endometrial cancer is strongly associated with obesity, and tumors often harbor mutations in major cancer signaling pathways. To inform the integration of body composition into targeted therapy paradigms, this hypothesis-generating study explores the association between muscle mass, body fat, and tumor proteomics. **Methods**: We analyzed data from 113 patients in The Cancer Genome Atlas (TCGA) and Cancer Proteomic Tumor Analysis Consortium (CPTAC) cohorts and their corresponding abdominal CT scans. Among these patients, tumor proteomics data were available for 45 patients, and 133 proteins were analyzed. Adiposity and muscle components were assessed at the L3 vertebral level on the CT scans. Patients were stratified into tertiles of muscle and fat mass and categorized into three groups: high muscle/low adiposity, high muscle/high adiposity, and low muscle/all adiposities. Linear and Cox regression models were adjusted for study cohort, stage, histology type, age, race, and ethnicity. **Results**: Compared with the high-muscle/low-adiposity group, both the high-muscle/high-adiposity (HR = 4.3, 95% CI = 1.0–29.0) and low-muscle (HR = 4.4, 95% CI = 1.3–14.9) groups experienced higher mortality. Low muscle was associated with higher expression of phospho-4EBP1(T37 and S65), phospho-GYS(S641) and phospho-MAPK(T202/Y204) but lower expression of ARID1A, CHK2, SYK, LCK, EEF2, CYCLIN B1, and FOXO3A. High muscle/high adiposity was associated with higher expression of phospho-4EBP1 (T37), phospho-GYS (S641), CHK1, PEA15, SMAD3, BAX, DJ1, GYS, PKM2, COMPLEX II Subunit 30, and phospho-P70S6K (T389) but with lower expression of CHK2, CRAF, MSH6, TUBERIN, PR, ERK2, beta-CATENIN, AKT, and S6. **Conclusions**: These findings demonstrate an association between body composition and proteins involved in key cancer signaling pathways, notably the PI3K/AKT/MTOR, MAPK/ERK, cell cycle regulation, DNA damage response, and mismatch repair pathways. These findings warrant further validation and assessment in relation to prognosis and outcomes in these patients.

## Simple Summary

Endometrial cancer, accounting for more than 90% of all cancers of the uterus body, is the most common gynecological cancer. Since 2005, new cases and deaths due to this cancer have steadily risen in the United States and worldwide, emphasizing the need for deeper insights into its underlying mechanisms to enhance prevention and improve patient outcomes. This exploratory study leveraged CT scans to accurately quantify muscle and adipose tissue. This study categorized patients into exclusive body composition types and examined the association between these body composition categories with protein expression and overall survival. The findings reveal significant survival differences among body composition types and associations with proteins involved in key cancer signaling pathways. Our findings warrant further validation in studies with large sample sizes and correct temporality, with a long-term goal of using body composition and tumor markers for risk stratification among patients with endometrial cancer.

## 1. Introduction

Endometrial cancer is the most common gynecological malignancy affecting women in the United States and ranks fourth in cancer incidence among women [1]. Within the United States, in 2024, it is anticipated that there will be 67,880 new cases and 13,250 deaths due to uterine corpus cancers, more than 90% of which are endometrial cancers [2]. Globally, endometrial cancer ranks sixth in incidence in women, with approximately 417,000 newly diagnosed cases and 97,370 deaths in 2020 [3]. Endometrial cancer mortality and incidence rates have been rising since 2005, with an annual percentage incidence change of 1.3% for the U.S. and 0.5% globally [1,4,5]. This necessitates a better understanding of the mechanisms underlying endometrial cancer for prevention and treatment.

Obesity is a significant risk factor for endometrial cancer and a determinant of the progression and treatment outcomes of this disease [6,7]. Obesity-induced inflammation, insulin resistance, and hormonal and metabolic alterations interact with cancer-signaling pathways [7]. The body mass index (BMI), expressed in kg/m^2^, is a convenient measure of body size and an indirect estimate of adiposity [8]. An ongoing systematic review highlights that studies investigating the relationship between body composition, its components, and tumor biomarker expression and associated pathways, have relied on the BMI as the primary measure for estimating body composition [9]. However, the BMI is unable to provide precise estimates of adipose tissue or skeletal muscle volume [8,10], especially because it disregards bone density and muscle mass. For example, excess adiposity can be present in patients with a normal BMI, while high or low skeletal muscle can be present in patients with obesity or overweight [11]. Additionally, the BMI does not inform how body fat is distributed, and since visceral and subcutaneous adipose tissues have different inflammatory and metabolic profiles, quantifying each separately is of great importance in predicting disease risk [12].

Computerized tomography (CT) scans offer a superior alternative to the BMI for estimating body composition and are widely used in clinical settings. CT scans can provide accurate estimates of body composition and detailed information on fat mass and skeletal muscle tissue mass compared to the BMI [11]. In particular, the third lumbar vertebra (L3) slice has gained considerable attention because of its ability to reflect whole-body composition when using the appropriate software [11].

Tumor signaling pathways are important indicators of endometrial cancer progression, response to therapy, and overall survival [13]. Nevertheless, the association between body compositions determined by CT scans and tumor signaling pathways in endometrial cancer remains unexplored. Studying this relationship would provide additional knowledge on the mechanisms that influence disease progression and treatment outcomes.

To fill the gap, this hypothesis-generating study aims to investigate the extent to which adipose and skeletal muscle quantities determined by CT scans are associated with protein expression in major tumor signaling pathways. We used publicly available data from patients with endometrial cancer via CT imaging and protein expression data in The Cancer Genome Atlas (TCGA) and Cancer Proteomic Tumor Analysis Consortium (CPTAC).

## 2. Materials and Methods

### 2.1. Study Participants

This study included patients from The Cancer Genome Atlas—Uterine Corpus Endometrial Carcinoma (TCGA-UCEC) and The Clinical Proteomic Tumor Analysis Consortium—Uterine Corpus Endometrial Carcinoma (CPTAC-UCEC) datasets. The Cancer Genome Atlas (TCGA) is a comprehensive, multi-institutional research project initiated by the National Cancer Institute (NCI) and the National Human Genome Research Institute (NHGRI), aimed at characterizing the genetic alterations underlying various cancer types, including endometrial cancer stored as TCGA-UCEC. It comprises vast data repositories, including clinical data, proteomic profiles of thousands of tumor samples across multiple cancer types, and radiology imaging of the corresponding patients. The clinical data are stored at the Genomic Data Commons (GDC), the imaging data repository is the Cancer Imaging Archive (TCIA), and the proteomic data repository is the Cancer Proteome Atlas (TCPA) [14].

The Clinical Proteomic Tumor Analysis Consortium (CPTAC), spearheaded by the National Cancer Institute, focuses on the proteomic characterization of tumors through mass-spectrometry-based analyses. It also provides data for patients with endometrial cancer (CPTAC-UCEC), including their clinical and proteomic data, found at the Proteomic Data Commons (PDC), and the corresponding radiology imaging data in TCIA [15].

Supplemental Figure S1 shows the flowchart of tumor protein selection. The TCGA-UCEC had 170 proteins assayed by reverse-phase protein array, while the CPTAC-UCEC had 12,247 proteins assayed by mass spectrometry. The 133 proteins present in both datasets were identified and used in the analysis stage. These proteins are listed in the Supplemental File S1.

Supplemental Figure S2 shows the flowchart of patient selection according to the proteomic data and CT scan availability. CT scan images were collected from standard-of-care imaging performed immediately before the pathological diagnosis or a follow-up image taken within three months from diagnosis. The TCGA-UCEC had 560 patients, and the CPTAC-UCEC had 240 patients. Only 140 patients had CT scans in the TCIA, 65 from the TCGA-UCEC, and 75 from the CPTAC-UCEC. In total, 13 patients from TCGA-UCEC and 14 from CPTAC-UCEC were excluded because they had ineligible CT scans defined as poor image quality, had scans with largely cropped L3 images with missing areas, or did not contain the L3 region. For patients with multiple CT scans, the earliest eligible scan was selected. L3 images from 113 patients were annotated for survival analyses. Subsequently, 22 patients from the TCGA-UCEC and 46 from the CPTAC-UCEC were excluded for missing tumor protein data or clinical data, resulting in the final sample of 45 patients, 30 patients from the TCGA-UCEC, and 15 from the CPTAC-UCEC, for proteomic data analyses.

### 2.2. CT Image Analysis

Abdominal cross-sectional fat and skeletal muscle areas from single-slice CT scan images at the L3 vertebra strongly correlate with the entire body’s adipose tissue and skeletal muscle mass volumes [16]. We selected an L3 image of the slice corresponding to this region for every patient using Sante DICOM Viewer Lite Version 3.1.5 (64 bit) (Santesoft LTD, Nicosia, Cyprus). The obtained image was then annotated, and surface areas were quantified using SliceOmatic, version 5.0 revision 8c (Tomovision, Montreal, QC, Canada). The annotated areas included visceral adipose tissue (VAT), subcutaneous adipose tissue (SAT), intramuscular adipose tissue (IMAT), and total skeletal muscle (TSM). The annotation was based on the Hounsfield units (HUs) and the anatomical location of the tissues. Supplemental Table S1 presents cutoff point values in Hounsfield units for each tissue [16]. All annotations were performed by a single researcher (C.M.M). Our study radiologist (X. N.) reviewed the protocol and all the annotated images.

The values of VAT, SAT, and IMAT were summed up to calculate the total adipose tissue (TAT) values. Based on TAT and VAT tertiles identified using the 45 patients without missing data (TAT cutoffs = 455 cm^2^ and 750.4 cm^2^, TSM cutoffs = 122.1 cm^2^ and 140.7 cm^2^), the patients were classified into three mutually exclusive body composition categories: (1) high muscle/low adiposity, defined as patients with the highest two tertiles of TSM and the lowest two tertiles of TAT; (2) high muscle/high adiposity, defined as those with the highest tertile of TAT and the highest two tertiles of TSM; and (3) low muscle, defined as those with the lowest tertile of TSM and any of the adiposity tertiles (Supplemental Table S2). Research using a similar approach showed that patients with colorectal cancer with low muscle/high adiposity were at increased risk of mortality compared to those with high muscle/low adiposity [17]. However, in our study, only one patient was in the low-muscle/high-adiposity category, so we combined the category into the low muscle group.

### 2.3. Statistical Analysis

The demographic and clinical characteristics were summarized as means and standard deviation for continuous variables and percentages for categorical variables. Pearson’s correlation coefficient (*r*) was calculated for the correlation between the BMI and TSM and between the BMI and TAT. Adjusted Kaplan–Meier curves and Cox Proportional Hazard models for overall survival analysis were performed with the high-muscle/low-adiposity group as the reference group for the 113 patients with satisfactory L3 CT images to estimate hazard ratios (HRs) and the corresponding 95% confidence intervals.

Multivariable linear regression analysis was performed to assess the association between body composition types and tumor protein expression in log2 normalized values, with high muscle/low adiposity as the reference group. Covariates included in the Cox and regression models were the study (whether TCGA-UCEC or CPTAC-UCEC), stage, histology type, race, ethnicity, and age. Adjusting the Cox model for the BMI may result in an over-adjusted HR because the BMI is highly correlated with total adipose tissue. Therefore, we only considered it as a sensitivity analysis.

To understand the utility of predicting tumor protein expression using the body composition type compared with BMI categories, we performed regression analysis for class I/II obesity (BMI 30.0–39.9) and class III obesity (BMI ≥ 40), compared with normal/overweight (BMI < 30.0) as the reference group. These BMI categories were derived from the WHO criteria [18]. We combined the normal weight and overweight groups because only a small (*n* = 5) number of patients had a normal weight. All tests were two-sided; *p*-value < 0.05 was considered statistically significant. False Discovery Rate (FDR) adjustment was performed using the Benjamini–Hochberg procedure. All statistical analyses were performed using SAS 9.4 (SAS Institute Inc., Cary, NC, USA). Volcano plots were generated using GraphPad Prism version 10.1.2 (GraphPad Software, Boston, MA, USA).

## 3. Results

### 3.1. Demographic Tumor Characteristics of Study Participants

Table 1 summarizes the demographic characteristics, histopathologic features, anthropometric characteristics, and body composition distributions of the patients with both image and tumor proteomics data. The median age was 63.3 years, ranging from 34 to 87 years. Most (86.7%) of the patients were white. The percentage of patients with stage I, II, and III was 51.1%, 13.3%, and 26.7%, respectively. Three-quarters (73.3%) of the patients had endometrioid histological carcinoma, and 26.7% had a serous type. Two-thirds (66.7%) of the patients had a stable microsatellite status, while a quarter (26.7%) of the patients had a high level of microsatellite instability, and 4.4% had a low level of microsatellite instability. The average BMI was 35.5 kg/m^2^, with 11.1% of the participants having a normal BMI, 15.6% in the overweight category, 20.0% in the class I obesity category, 26.7% in the class II obesity category, and 26.7% with class III obesity. In the normal/overweight group, 83.3% of the patients were in the low-muscle category (Supplemental Table S3).

### 3.2. Body Composition Characteristics of Study Participants

**Figure 1:**
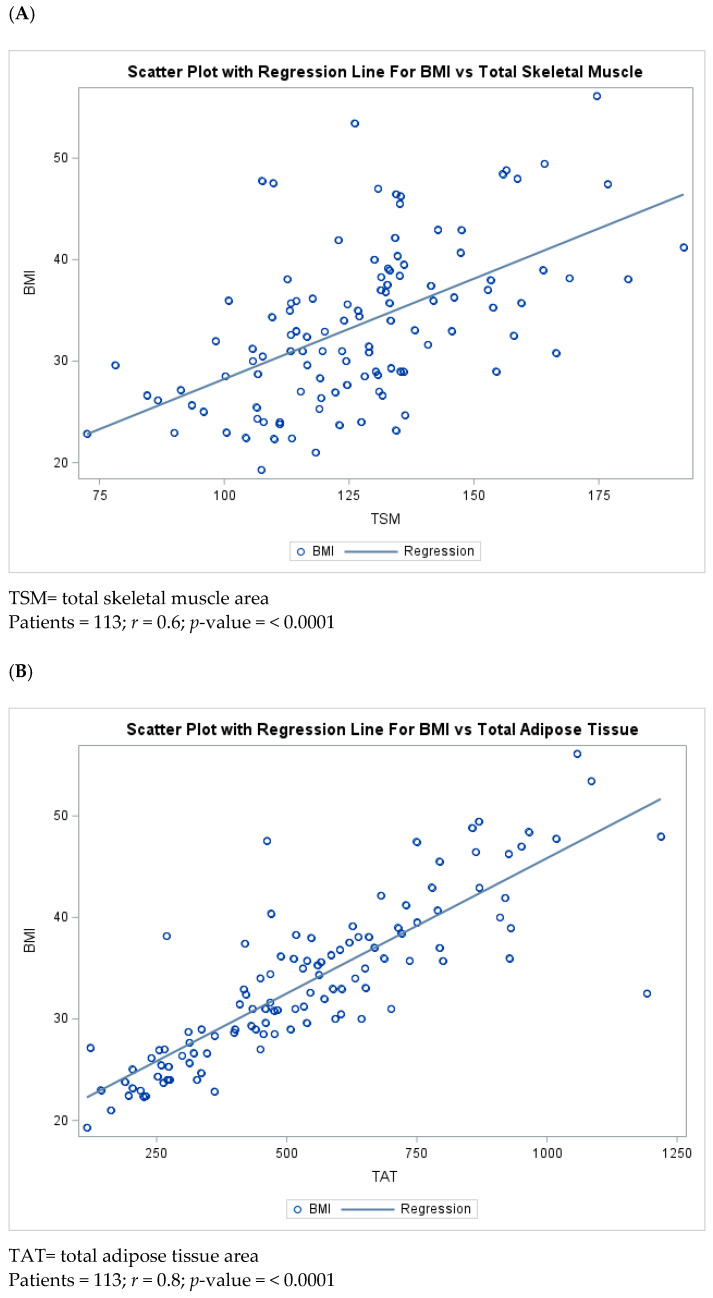
Correlation between TSM (**A**) and TAT (**B**) with the BMI of the study participants.

**Figure 2:**
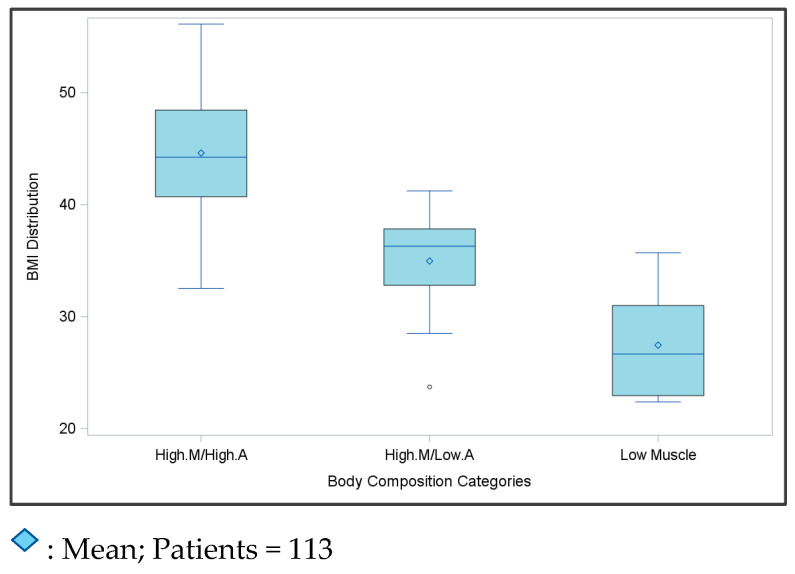
BMI distribution across the body composition groups of the study participants.

The correlation of the BMI with TAT and TSM are shown in Figure 1 (TSM Pearson’s *r* = 0.6 and TAT Pearson’s *r* = 0.8, both with a *p*-value = <0.0001). On average, there was a larger variability in the BMI in TSM compared to TAT. Figure 2 shows how the BMI is distributed across all body composition categories. Supplemental Figure S3 shows the mean areas of each body composition component by the body composition type. Patients in the low-muscle category tended to have the lowest average TSM, SAT, and VAT, while patients with high muscle/low adiposity had the lowest average IMAT but the highest average TSM area.

**Figure 3:**
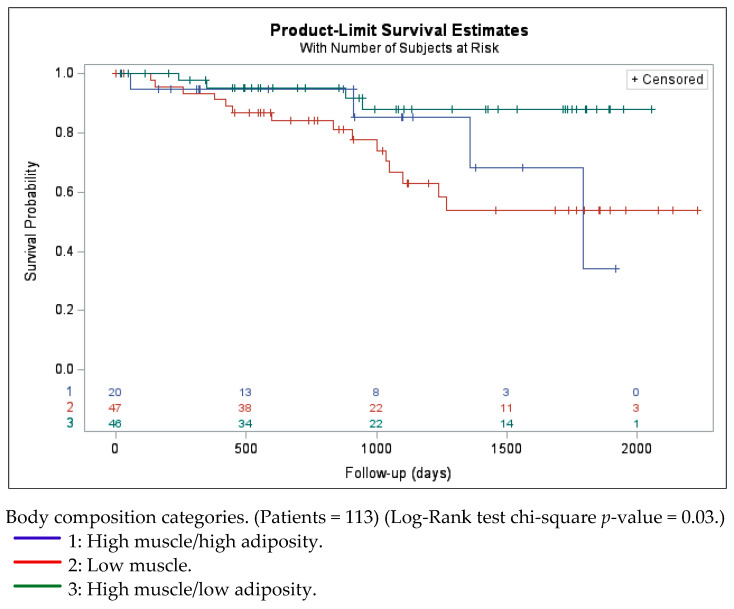
Kaplan–Meier graph exploring the survival trend based on body composition group.

The low-muscle group experienced the worst outcome (HR, 4.4; 95% CI, 1.3–14.9; *p*-value = 0.02) compared to the reference group. Similarly, the survival analysis revealed that patients in the high-muscle/high-adiposity group also had a more than four-fold increased risk of mortality compared to those with high muscle/low adiposity (HR, 4.3; 95% confidence interval [CI], 1.0–19.0; *p*-value = 0.05). (Table 2; Figure 3). Histological subtype (endometrioid vs. serous) did not demonstrate a significant association with overall survival outcomes. However, a more advanced stage was associated with a higher mortality risk (Supplemental Table S4).

### 3.3. Tumor Protein Expression Across Body Composition Categories

**Figure 4:**
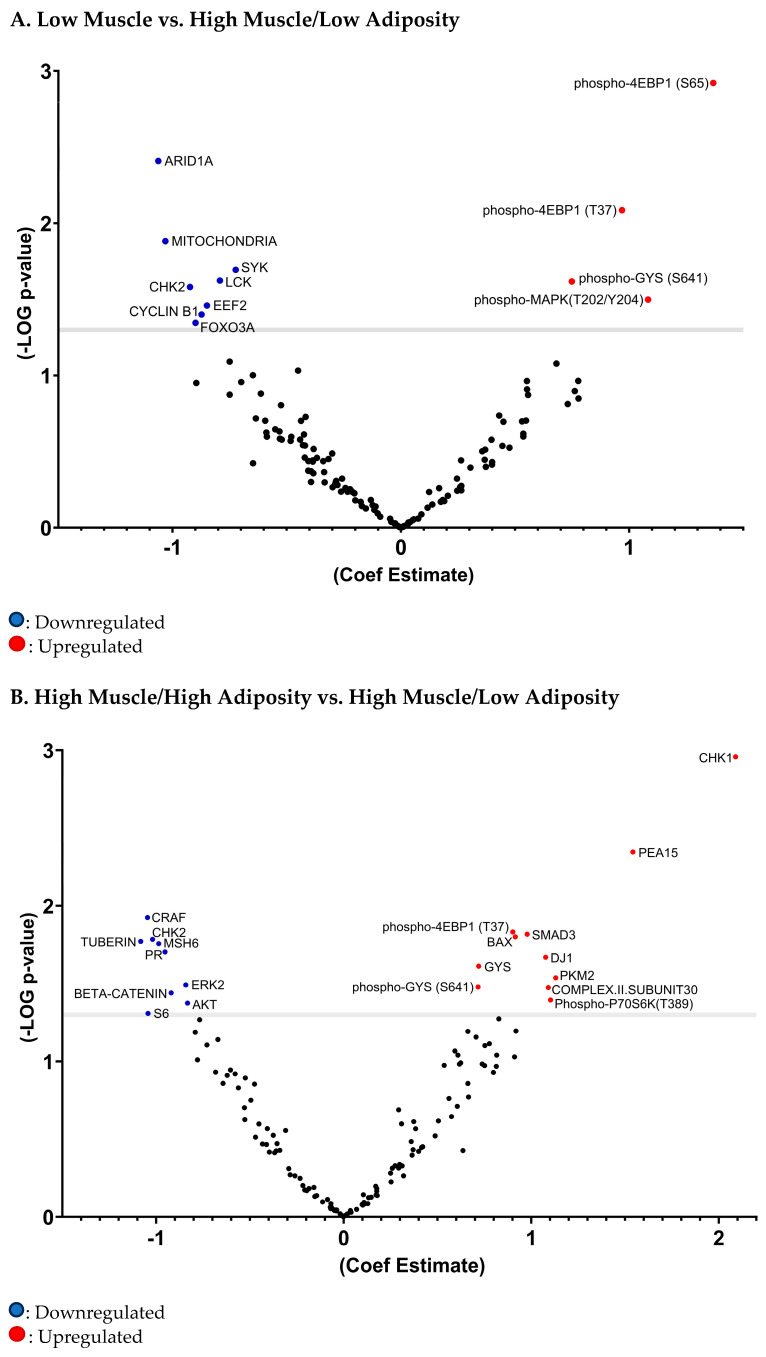
Volcano plot of differential expression of protein tumors based on body composition groups with high muscle/low adiposity as a referent group.

In the regression analysis model for tumor protein expression comparing body composition types (Table 3; Figure 4A; Supplemental File S1), the low-muscle (vs. high-muscle/low-adiposity) body composition type was associated with higher expression of phosphorylated eukaryotic translation initiation factor 4E-binding protein 1 at threonine 37 (phospho-4EBP1(T37)) (log2-fold change = 1.0, *p*-value = 0.008), phosphorylated eukaryotic translation initiation factor 4E-binding protein 1 at serine 65 (phospho-4EBP1(S65)) (log2-fold change = 1.4, *p*-value = 0.001), phosphorylated mitogen-activated protein kinase at threonine 202 and tyrosine 204 (phospho-MAPK(T202/Y204) (log2-fold change = 1.1, *p*-value = 0.001) and phosphorylated glycogen synthase at serine 641 (phospho-GYS(S641)) (log2-fold change = 0.7, *p*-value = 0.024) but lower expression of AT-rich interactive domain-containing protein 1 (ARID1A) (log2-fold change = −1.1, *p*-value= 0.004), checkpoint kinase 2 (CHK2) (log2-fold change = −0.9, *p*-value = 0.026), spleen Tyrosine Kinase (SYK) (log2-fold change = −0.7, *p*-value = 0.020), lymphocyte-specific protein tyrosine kinase (LCK) (log2-fold change = −0.8, *p*-value = 0.024), eukaryotic elongation factor 2 (EEF2) (log2-fold change = −0.8, *p*-value = 0.035), forkhead box O3 (FOXO3A) (log2-fold change = −0.9, *p*-value = 0.045) and cyclin B1 (log2-fold change = −0.9, *p*-value = 0.040).

The high muscle/high adiposity (vs. high muscle/low adiposity) body composition type was associated with higher expression of phospho-4EBP1 (T37) (log2-fold change = -0.9, *p*-value = 0.015), GYS (log2-fold change = 0.7, *p*-value = 0.024), phospho-GYS (S641) (log2-fold change = 0.7, *p*-value = 0.033), checkpoint kinase 1 (CHK1) (log2-fold change = 2.1, *p*-value = 0.001), proliferation and apoptosis adaptor protein 15 (PEA15) (log2-fold change = 1.5, *p*-value = 0.005), mothers against decapentaplegic homolog 3 (SMAD3) (log2-fold change = 1.0, *p*-value = 0.015), Bcl-2-associated X protein(BAX) (log2-fold change = 0.9, *p*-value = 0.016), DJ-1 (log2-fold change = 1.1, *p*-value = 0.021), Pyruvate Kinase Muscle Isozyme 2 (PKM2) (log2-fold change = 1.1, *p*-value = 0.029), complex II subunit 30 also known as succinate dehydrogenase complex (log2-fold change = 1.1, *p*-value = 0.033), and phosphorylated P70S6 kinase at threonine 389 (phospho-P70S6K (T389) (log2-fold change = 1.1, *p*-value = 0.040) but with lower expression of CHK2 (log2-fold change = −1.0, *p*-value = 0.016), CRAF (Raf-1 proto-oncogene) (log2-fold change = −1.0, *p*-value = 0.012), MutS homolog 6 (MSH6) (log2-fold change = −1.0, *p*-value = 0.018), Progesterone receptor (PR) (log2-fold change = −1.0, *p*-value = 0.020), Tuberin (log2-fold change = −1.1, *p*-value = 0.017), extracellular signal-regulated kinase 2 (ERK2) (log2-fold change = −0.8, *p*-value = 0.032), beta-catenin (log2-fold change = −0.9, *p*-value = 0.036), AKT, also known as Protein Kinase B (log2-fold change = −0.8, *p*-value = 0.042), and S6 (log2-fold change = −1.0, *p*-value = 0.049) (Table 3; Figure 4B; Supplemental File S1).

### 3.4. Tumor Protein Expression Across BMI Categories

The adjusted regression analysis model for tumor protein expression comparing BMI-based groups showed that the class I/II obesity (vs. normal/overweight) group was significantly associated with higher expression of fibronectin (log2-fold change = 0.7, *p*-value = 0.034), Tumor Protein P53 Binding Protein 1 (TP53BP1; log2-fold change = 0.7, *p*-value = 0.043), and Carbonic Anhydrase 9 (CA9; log2-fold change = 0.9, *p*-value = 0.043) but lower expression of phospho-S6 (S240/S244) (log2-fold change = −0.7, *p*-value = 0.038) (Supplemental Table S5 and Supplemental Figure S4A). The class III obesity (vs. normal/overweight) group was significantly associated with higher expression of CHK1 (log2-fold change = 1.4, *p*-value = 0.031) and Beclin 1 (log2-fold change = 0.6, *p*-value = 0.047) but lower expression of androgen receptor (AR; log2-fold-change = −0.8, *p*-value = 0.039) (Supplemental File S1; Supplemental Figure S4B).

## 4. Discussion

Considering the variation in overall survival outcomes across the identified body composition categories, it is crucial to understand the differences in the underlying tumor molecular expressions. To our knowledge, this is the first study to investigate the association between CT-scan-assessed body composition components, including adipose and skeletal muscle tissue, with endometrial tumor protein expression. All studies we reviewed during the preliminary literature review for this study employed the BMI as a measure of adiposity, body size, or body composition estimate. After adjusting for potential confounders, body composition types were associated with tumor expression of several proteins that are commonly found in key endometrial cancer signaling pathways, including the PI3K/AKT/MTOR, DNA damage response, and mismatch repair pathways.

Our analysis indicated that the low-muscle group and the high-muscle/high-adiposity group (vs. high muscle/low adiposity) showed significantly elevated levels of phospho-4EBP1 (T37 and S65) and phospho-GYS (S641), alongside reduced ARID1A expression for the low-muscle/all adiposities group. The translation repressor and tumor suppressor 4EBP1 is phosphorylated by MTOR Complex 1 [19]. Phosphorylation of 4EBP1 deactivates the enzyme’s tumor suppressor function, and elevated phospho-4EBP1 levels are associated with worse survival in patients with cancer [20,21,22]. Additionally, GYS1 (the muscle isoform of glycogen synthase) catalyzes the rate-limiting step of glycogen biosynthesis, regulated by pathways involving kinases like AMPK and GSK-3 [23]. Similarly, the phosphorylation of GYS1 makes the enzyme inactive [24]. This dysregulation, common in cancers, contributes to altered metabolism, promoting cancer progression, including increased tumor glycolytic activity (Warburg effect) and glycogen synthase inactivation [25].

Our analysis of the high-muscle/high-adiposity body composition type is consistent with our current understanding that adiposity promotes tumorigenesis through IGF/insulin signaling, inflammation, and estrogen, leading to DNA instability [7,26]. Importantly, the upregulation of phospho-4EBP1 and phospho-GYS—implying their inactivation—in both the low-muscle/all adiposities and high-muscle/high-adiposity groups, in addition to phospho-P70S6K (T389) for the high-muscle/high-adiposity group, suggests upregulated PI3K/AKT/MTOR signaling. This upregulation may partly explain the reduced survival observed in these two groups compared to the high-muscle/low-adiposity body composition group. Various studies reported that this signaling pathway negatively affects survival through various mechanisms. The mechanisms include inhibiting apoptosis by upregulating anti-apoptotic genes such as BCL-2, therefore protecting cancerous cells from programmed cell death and enhancing various transporters and proteins that lead to multidrug resistance in cancer [27,28,29]. Our analyses suggested that both high adiposity (high muscle/high adiposity vs. high muscle/low adiposity) and low muscle are associated with higher PI3K/AKT/MTOR signaling. This suggests that increased muscle mass and lowered adiposity may effectively tame the signaling pathway and lead to pro-survival signaling.

As stated above, the low-muscle group had downregulated ARID1A protein compared to the high-muscle/low-adiposity body composition type. ARID1A is a subunit of the BAF chromatin remodeling complex that facilitates chromatin remodeling critical during development and serves as a tumor suppressor. The ARID1A gene is commonly mutated or lost in ovarian clear-cell and endometrioid carcinomas and pre-cancer lesions [30,31,32,33,34]. The loss of ARID1A expression is related to shorter progression-free survival [35]. The downregulation of ARID1A in our study is consistent with upregulated mTOR signaling. Previous studies reported that ARID1A loss activates mTOR signaling [36], and another study reported that ARID1A loss is associated with AKT phosphorylation [37].

Additionally, the downregulation of other proteins that have a role in endometrial and other cancers, including SYK, FOXO3A, and cyclin B1, offers insights into the worst survival of the low-muscle/all adiposities group compared to the others [38,39,40,41]. Research has also shown that SYK expression in some solid tumors is associated with increased tumor-infiltrating lymphocytes and better survival [42]; therefore, its decreased expression hints at a potentially affected immune response within the tumor microenvironment and a poorer prognosis. Similarly, the downregulation of FOXO3A likely enhances cancer cell survival and resistance to apoptosis, while the downregulation of cyclin B1 suggests a possible defect in cell cycle regulation, both affecting the prognosis of these patients compared to the patients in the high-muscle/low-adiposity group [40,43].

Furthermore, this study found that body composition also modulated checkpoint kinase protein levels. For example, checkpoint kinase 1 (CHK1) was elevated among the high-muscle/high-adiposity group (vs. high muscle/low adiposity). This protein responds to DNA damage or cytotoxic stress by arresting the cell cycle until damaged DNA has been repaired. However, in doing so, it may also promote tumor progression by maintaining the genomic stability of cancerous cells [44,45]. Thus, the high oxidative stress and the resulting DNA damage that comes with high muscle/high adiposity may have contributed to CHK1 upregulation. On the other hand, the CHK2 protein was downregulated in both the low-muscle and the high-muscle/high-adiposity groups compared to the high-muscle/low-adiposity body composition type. The CHK2 protein is encoded by *CHEK2*, a gene that is frequently mutated in endometrial cancer [46]. Similarly to CHK1, CHK2 regulates DNA repair and metastasis inhibition. However, research on obesity and CHK1/2 is very limited and warrants investigation.

Complex II subunit 30 (succinate dehydrogenase complex) was also upregulated among the high-muscle/high-adiposity (vs. high-muscle/low-adiposity) group. The protein is important for cell growth and metabolism and is involved in the citric acid cycle (Krebs cycle) and the electron transport chain [47]. Its upregulation might suggest altered mitochondrial function and energy metabolism in tumors related to high adiposity. SMAD3 upregulation suggests alterations in TGF-β signaling [48,49]. This pathway is involved in cellular processes, including cell growth, differentiation, and apoptosis, and its dysregulation contributes to tumor progression [49]. In endometrial cancer, the inhibition of this pathway has been shown to suppress metastasis, making it a potential target for intervention among patients in this category [50]. The high muscle/high adiposity body composition type also exhibited the downregulation of CRAF, ERK2, beta-catenin, Tuberin (TSC2), S6, and AKT proteins, which collectively suggest that the high adiposity in endometrial cancer is associated with alterations in the MAPK and PI3K/AKT/MTOR pathways, both of therapeutic potential in endometrial cancer [51,52]. The downregulation of beta-catenin, a component of the Wnt/beta-catenin signaling pathway, in tumors of patients with high muscle mass and high adiposity presents an intriguing contradiction to the existing literature and the expected prognosis. Previous studies on muscle in animal models and adipocyte precursor cells demonstrated that beta-catenin depletion reduces muscle mass and inhibits adipogenesis, respectively [53,54]. Notably, WNT/beta-catenin upregulation is associated with the progression of endometrial cancer, with many studies evaluating its potential as a therapeutic target [55,56]. This unexpected finding highlights the complex relationship between body composition and tumor biology, underlining the need for further investigation into the role of muscle mass and adiposity in modulating the Wnt/β-catenin pathway in the context of endometrial cancer and patient outcomes. The upregulation of PEA-15 (phospho-enriched protein in astrocytes), which exhibits both tumor-suppressor and -promoting properties, warrants further investigation to understand the relevance of this expression pattern in endometrial cancer [57].

The downregulation of PR (progesterone receptor), and MSH6 are consistent with the observation that high muscle/high adiposity is associated with worse outcomes among patients with endometrial cancer. Moreover, PR plays a major role in antagonizing the proliferative effects of estrogens in the endometrium. Its downregulation can lead to endometrial hyperplasia, potentially progressing to cancer [58]. MSH6 is a DNA mismatch repair protein that functions as mismatch recognition [59]. Suppression of these proteins, along with the activation of other proteins, including CHK1 by high adiposity, suggests novel mechanisms that underlie the adverse effect of high adiposity on endometrial cancer.

The utility of using the BMI to indicate proteins for understanding pathway signaling or therapeutic targets may be inferior to CT-assessed adiposity levels. It was not feasible to compare patients with obesity to those with normal weight because only five patients had normal weight. Thus, we combined the normal weight with the overweight groups to ensure sufficient sample sizes for robust statistical analysis. Nevertheless, most (83.3%) normal/overweight patients fell into the low-muscle body composition type, while the majority (61.9%) of patients with class I/II obesity were, in fact, in the high-muscle/low-adiposity category (Supplemental Table S3). The finding that 61.9% of class I/II obese patients were categorized as having high muscle mass and low adiposity is noteworthy. While this observation is primarily influenced by the predominance of patients in the obesity group in our sample, it also highlights the limitations of the body mass index (BMI) as a measure of body composition. The BMI can lead to the misclassification of individuals, particularly those with high muscle mass who can be categorized as obese, even if they have relatively low body fat. This limitation of the BMI underscores the need to use more accurate measures, especially in clinical settings, where it can have significant implications for clinical outcomes. The comparison of BMI-based categories only revealed a few markers potentially up- or downregulated by class I/II obesity. Additionally, the comparison between class III obesity and normal/overweight groups showed increased CHK1 expression, consistent with findings from the comparison of high-muscle/high-adiposity and high-muscle/low-adiposity body composition types. These results suggest that when studying obesity-driven signaling pathways in endometrial cancer, it may be necessary to consider body composition components to describe the influence of obesity more accurately.

The limitations of this study should be noted. The study was cross-sectional, as body composition was derived from CT scans performed around the time of tumor specimen collection. The small sample size limits the study’s statistical power, and hence this study should be regarded as hypothesis generating. Compared with all the patients in the TCGA-UCEC and CPTAC-UCEC study, the patients with CT images and tumor proteomics data were more likely to have a more advanced stage of disease and a higher BMI (Supplemental Table S6). Additionally, when comparing patients with CT scan images and proteomics data vs. all those with CT scan images, the former group tended to be younger and have a higher BMI. Moreover, there is a potential systematic error due to differences in measurement techniques of the protein expression data. We accounted for the effect of the differences in study cohorts in the regression models to reduce the potential bias due to this systematic error. Also, the number of proteins analyzed was limited by the relatively small RPPR panel. Therefore, we might have missed proteins with distinct features in the tumor and stroma components related to body composition.

## 5. Conclusions

In conclusion, this study is the first to provide insights into the association between CT-scan-assessed body composition and tumor protein expression in endometrial cancer. The findings suggest that both adiposity and muscle mass may influence critical signaling pathways in endometrial cancer tumors, with potential implications for targeted therapies. Findings from our study warrant further validation in studies with large sample sizes and correct temporality to ultimately use body composition and tumor markers for risk stratification among patients with endometrial cancer.

## Figures and Tables

**Table 1:** Summary of description statistics (Mean (SD) or No. (%)).

Characteristic	CPTAC-UCEC	TCGA-UCEC	Total
Number of patients	15 (33.3%)	30 (66.7%)	45
Age at diagnosis	64.8 (11.4)	62.5 (11.0)	63.3 (11.1)
Race			
White	10 (66.7%)	29 (96.7%)	39 (86.7%)
Black	1 (6.7%)	1 (3.3%)	2 (4.4%)
Missing	4 (26.7%)	0	4 (8.9%)
AJCC Pathological Stage			
Stage I	7 (46.7%)	16 (53.3%)	23 (51.1%)
Stage II	2 (13.3%)	4 (13.3%)	6 (13.3%)
Stage III	5 (33.3%)	7 (23.3%)	12 (26.7%)
Stage IV	1 (6.7%)	3 (10.0%)	4 (8.9%)
Ethnicity			
Not Hispanic or Latino	9 (60.0%)	29 (96.7%)	38 (84.4%)
Not reported	6 (40.0%)	1 (3.3%)	7 (15.6%)
Microsatellite instability (MSI) Status			
Stable	12 (80.0%)	18 (60.0%)	30 (66.7%)
High	3 (20.0%)	9 (30.0%)	12 (26.7%)
Low	0	2 (6.7%)	2 (4.4%)
Indeterminant	0	1 (3.3%)	1 (2.2%)
Histologic Type			
Serous	4 (26.7%)	8 (26.7%)	12 (26.7%)
Endometrioid	11 (73.3%)	22 (73.3%)	33 (73.3%)
BMI, continuous variable (kg/m^2^)	35.8 (7.7)	35.3 (9.0)	35.5 (8.5)
BMI Classification			
Normal (18.5–<25.0)	1 (6.7%)	4 (13.3%)	5 (11.1%)
Overweight (25.0–<30.0)	3 (20.0%)	4 (13.3%)	7 (15.5%)
Class I obesity (30.0–<35.0)	3 (20.0%)	6 (20.0%)	9 (20.0%)
Class II obesity (35.0–<40.0)	4 (26.7%)	8 (26.7%)	12 (26.7%)
Class III obesity (≥40.0)	4 (26.7%)	8 (26.7%)	12 (26.7%)
Body composition			
High muscle/Low adiposity	5 (33.3%)	11 (36.7%)	16 (35.6%
High muscle/High adiposity	5 (33.3%)	9 (30.0%)	14 (31.1%)
Low muscle	5 (33.3%)	10 (33.3%)	15 (33.3%)

**Table 2:** Hazard ratios of mortality for body composition.

	Body Composition Type (Patients = 113, Deaths = 23)		
	High muscle/ Low adiposity	High muscle/ High adiposity	Low muscle
Number of patients	46	20	47
Deaths	4	4	15
Crude HR (95% CI)	1.0 (ref.)	2.8 (0.7–11.3)	4.0(1.3–12.0)
Adjusted HR (95% CI) ^a^	1.0 (ref.)	4.3 (1.0–19.0)	4.4 (1.3–14.9)
Adjusted HR (95% CI) ^b^	1.0 (ref.)	2.5 (0.4–14.9)	5.7 (1.5–21.8)

HR = Hazard Ratio. CI = Confidence Interval. ^a^ Adjusted for study group, histology type, stage, age, race, and ethnicity. ^b^ Adjusted for study group, histology type, stage, age, race, ethnicity, and BMI.

**Table 3:** Differential protein expression in endometrial cancer tissue according to body composition types.

	Low Muscle vs. High Muscle/Low Adiposity			High Muscle/High Adiposity vs. High Muscle/Low Adiposity		
Protein Name	Estimate	*p*-Value	False Discovery	Estimate	*p*-Value	False Discovery
phospho-4EBP1 (S65)	1.4	0.001 *	0.207	0.6	0.102	0.406
phospho-MAPK(T202/Y204)	1.1	0.032 *	0.498	0.5	0.301	0.624
phospho-4EBP1 (T37)	1.0	0.008 *	0.446	0.9	0.015 *	0.298
phospho-GYS (S641)	0.7	0.024 *	0.495	0.7	0.033 *	0.335
SYK	−0.7	0.02 *	0.495	0.0	0.901	0.953
LCK	−0.8	0.024 *	0.495	−0.2	0.646	0.872
EEF2	−0.8	0.035 *	0.498	−0.6	0.123	0.418
CYCLIN B1	−0.9	0.04 *	0.511	−0.8	0.065	0.406
FOXO3A	−0.9	0.045 *	0.511	−0.1	0.799	0.943
CHK2	−0.9	0.026 *	0.495	−1.0	0.016 *	0.298
ARID1A	−1.1	0.004 *	0.332	0.0	0.956	0.973
CHK1	−0.4	0.501	0.819	2.1	0.001 *	0.188
PEA15	−0.4	0.422	0.804	1.5	0.005 *	0.298
PKM2	−0.4	0.438	0.81	1.1	0.029 *	0.335
Phospho-P70S6K(T389)	0.8	0.142	0.708	1.1	0.04 *	0.359
COMPLEX II SUBUNIT30	−0.3	0.542	0.819	1.1	0.034 *	0.335
DJ1	0.0	0.946	0.993	1.1	0.021 *	0.304
SMAD3	−0.2	0.559	0.819	1.0	0.015 *	0.298
BAX	0.0	0.986	0.993	0.9	0.016 *	0.298
GYS	−0.3	0.325	0.749	0.7	0.024 *	0.319
AKT	0.0	0.913	0.993	−0.8	0.042 *	0.359
ERK2	0.1	0.88	0.993	−0.8	0.032 *	0.335
beta-CATENIN	0.0	0.977	0.993	−0.9	0.036 *	0.343
PR	−0.1	0.762	0.913	−1.0	0.02 *	0.304
MSH6	−0.4	0.288	0.735	−1.0	0.018 *	0.298
S6	−0.6	0.252	0.713	−1.0	0.049 *	0.379
CRAF	−0.4	0.264	0.713	−1.0	0.012 *	0.298
TUBERIN	0.0	0.974	0.993	−1.1	0.017 *	0.298

Only associations with *p*-value < 0.05 in either of the adjusted models are listed. Models were adjusted for study cohort, cancer stage, histological type, race, ethnicity, and age. *: *p*-value < 0.05.

## Data Availability

The CT images are available at the Cancer Imaging Archive (TCGA: https://www.cancerimagingarchive.net/collection/tcga-ucec/ (accessed on 7 August 2023), CPTAC: https://www.cancerimagingarchive.net/collection/cptac-ucec/ (accessed on 7 August 2023)); clinical and protein data are available at the National Cancer Institute’s Proteomic Data Commons (for CPTAC-UCEC: https://pdc.cancer.gov/pdc/browse/filters/primary_site:Uterus,%20NOS, accessed on 7 August 2023) and Genomic Data Commons (for TCGA-UCEC: https://portal.gdc.cancer.gov/projects/TCGA-UCEC, accessed on 7 August 2023). Data generated by the authors are available on request.

## Supplementary Materials

The following supporting information can be downloaded at https://www.mdpi.com/article/10.3390/cancers16244222/s1, File S1: Protein list and Regression analysis results; Figure S1: Tumor Protein Selection; Figure S2: Patient selection flow chart; Figure S3: Total muscle and adipose tissue distribution within the body composition types; Figure S4: Volcano plot of differential expression of protein tumors based on BMI categories with normal/overweight as a referent group; Table S1: Tissue radiodensity cutoff points in Hounsfield units for CT image annotation; Table S2: Body composition categorization based on total muscle and adiposity tertiles; Table S3: BMI-based categories and the distribution of body composition types; Table S4: Cox proportional hazards model results for all variables; Table S5: Differential gene expressions in obesity class I/II and obesity class III relative to normal/overweight BMI; Table S6: Characteristics of patients by the availability of CT images and tumor proteomics data.
