# Supplementary material for: CT-Scan-Assessed Body Composition and Its Association with Tumor Protein Expression in Endometrial Cancer: The Role of Muscle and Adiposity Quantities
Source: Cancers (Basel). 2024 Dec 18;16(24):4222. doi: 10.3390/cancers16244222 (PMC11674723; doi:10.3390/cancers16244222)
Supplement: Supplementary file 1 [file cancers-16-04222-s001.zip › Supplemental tables and figures.pdf]

Supplemental tables and figures.

Supplemental Table S1: Tissue radiodensity cutoff points in Hounsfield units for CT image annotation.

**Tissue**	**Localization sites of tagged tissues**	**Hounsfield unit, lower limit**	**Hounsfield unit, upper limit**
Subcutaneous adipose tissue (SAT)	Subcutaneous fat	-190	-30
Intermuscular Adipose tissue (IMAT)	Inter-muscular fat	-190	-30
Visceral adipose tissue (VAT)	Fat around organs	-150	-50
Total skeletal muscle	Skeletal muscle	-29	200
Bone	Bone	200	2001

Supplemental Table S2: Body composition categorization based on total muscle and adiposity tertiles.

<table>
<tr><th></th><th>Total muscle tertile 1 (lowest)</th><th>Total muscle tertile 2</th><th>Total muscle tertile 3 (highest)</th></tr>
<tr><td>Total adiposity tertile 1 (lowest)</td><td rowspan="3">Low muscle</td><td colspan="2" rowspan="2">High muscle/Low adiposity</td></tr>
<tr><td>Total adiposity tertile 2</td></tr>
<tr><td>Total adiposity tertile 3 (highest)</td><td colspan="2">High muscle/High adiposity</td></tr>
</table>

Supplemental Table S3: BMI-based categories and the distribution of body composition types. The numbers are shown as N and row percentages.

**BMI-based category**	**Total**	High muscle/Low adiposity	High muscle/High adiposity	Low muscle
Normal/Overweight (< 30.0)	12	2 (16.7%)	0	10 (83.3%)
Obesity class I/II (30.0 – 39.9)	21	13 (61.9%)	3 (14.3%)	5 (23.8%)
Obesity class III (≥ 40)	12	1 (8.3%)	11 (91.7%)	0 (%)

Supplemental Table S4: Cox proportional hazards model results for all variables.

Variable	Hazard Ratio	95% Confidence Interval
**Body Composition**		
High muscle/Low adiposity	1.0 (ref.)	
High muscle/High adiposity	4.3	(1.0-19.0)
Low muscle	4.4	(1.3-14.9)
**Histology**		
Serous	1.0 (ref.)	
Endometrioid	1.4	(0.4,5.3)
**Study cohort**		
CPTAC-UCEC	1.0 (ref.)	
TCGA-UCEC	16.5	(1.9,141.7)
**AJCC pathological stage**		
Stage I	1.0 (ref.)	
Stage II	3.5	(0.6, 19.8)
Stage III	13.5	(3.3, 55.3)
Stage IV	9.3	(2.1, 41.0)
**Race**		
White	1.0 (ref.)	
Black or African American	0	.
American Indian or Alaska Native	16.3	(0.7, 386.3)
Other	0	.
**Ethnicity**		
Hispanic or Latino	1.0 (ref.)	
Not Hispanic or Latino	1.8	(0.2,19.7)
Not reported	8.0	(0.3, 201.9)
**Age**	1.1	(1.1,1.2)

AJCC: American Joint Committee on Cancer

Supplemental Table S5: Differential gene expressions in obesity class I/II and obesity class III relative to normal/overweight BMI.

Protein	**Obesity class I/II vs. Normal/Overweight**		**Obesity class III vs. Normal/Overweight**	
	**Estimate**	**P value**	**Estimate**	**P value**
TP53BP1	0.7	0.043*	0.6	0.185
CA9	0.9	0.043*	0.5	0.304
FIBRONECTIN	0.7	0.034*	0.2	0.577
Phospho-S6 (S240/S244)	-0.7	0.038*	-0.6	0.113
CHK1	-0.2	0.686	1.4	0.031*
AR	-0.2	0.610	-0.8	0.039*
BECLIN	0.1	0.584	0.6	0.047*

Only proteins with differential expressions with a p-value <0.05 in the adjusted model are included. The model was adjusted for the study cohort, cancer stage, and histological type.
**: p-value <0.05.*

Supplemental Table S6: Characteristics of patients by the availability of CT images and tumor proteomics data.

	Patients with CT images and tumor proteomics data	Patients with CT images	All patients
**Characteristics**	**Mean (SD) or No. %**	**Mean (SD) or No. %**	**Mean (SD) or No. %**
**N**	45	113	780
Age at diagnosis (years)	57.1 (10.7)	65.1 (10.6)	64.2 (10.7)
**Race**			
White	39 (86.7%)	100 (88.5%)	550 (70.5%)
Black	2 (4.4%)	7 (6.2%)	115 (14.7%)
Asian	0	0	23 (2.9%)
N. Hawaiian/ P. Islander	0	0	8 (1.0%)
A. Indian/ A. Native	0	0	4 (0.5%)
Others	0	1 (0.9%)	45 (5.7 %)
Missing	4 (8.9%)	4 (3.5%)	35 (4.5%)
**Ethnicity**			
Hispanic or Latino	7 (15.6%)	2 (1.8%)	23 (2.9%)
Not Reported	38 (84.4%)	70 (62.0%)	471 (60.4%)
Missing	0	41 (36.3%)	286 (36.7%)
**AJCC pathological stage**			
I	23 (51.1%)	64 (56.6%)	509 (65.3 %)
II	6 (13.3%)	14 (12.4%)	72 (9.2 %)
III	12 (26.7%)	25 (22.1%)	159 (20.4 %)
IV	4 (8.9%)	10 (8.9%)	40 (5.1%)
**BMI, continuous variable (Kg/m^2)**	35.5 (8.5)	33.6 (7.8)	34.0 (11.8)
**BMI categories**			
Normal weight (18.5-24.9)	5 (11.1%)	16 (14.1%)	123 (15.8 %)
Overweight (25 – 29.9)	7 (15.6%)	25 (22.1%)	160 (20.5%)
Obesity (> = 30)	33 (73.0%)	72 (63.7%)	466 (59.7%)

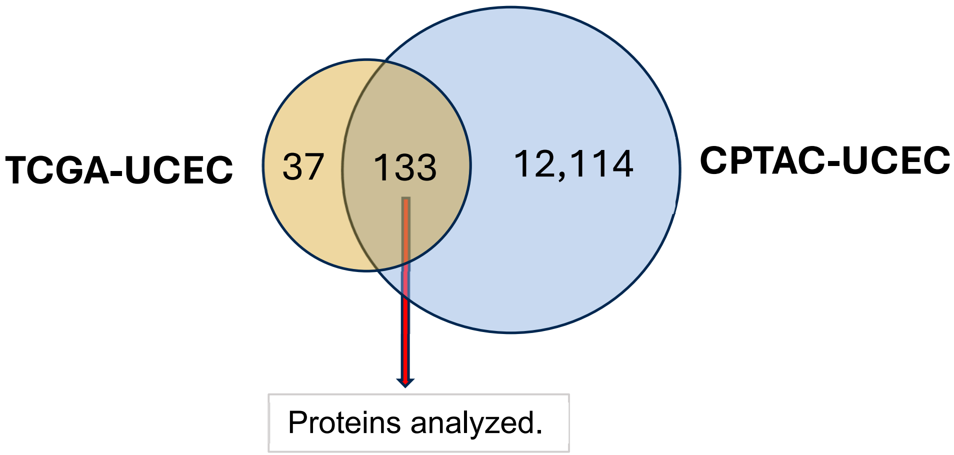

Supplemental Figure S1: Tumor Protein Selection.

Only proteins present in both datasets were used in the analysis, including proteins stored under a different name in CPTAC but representing the same protein; for example, CD31 is named PECAM1 in CPTAC-UCEC.

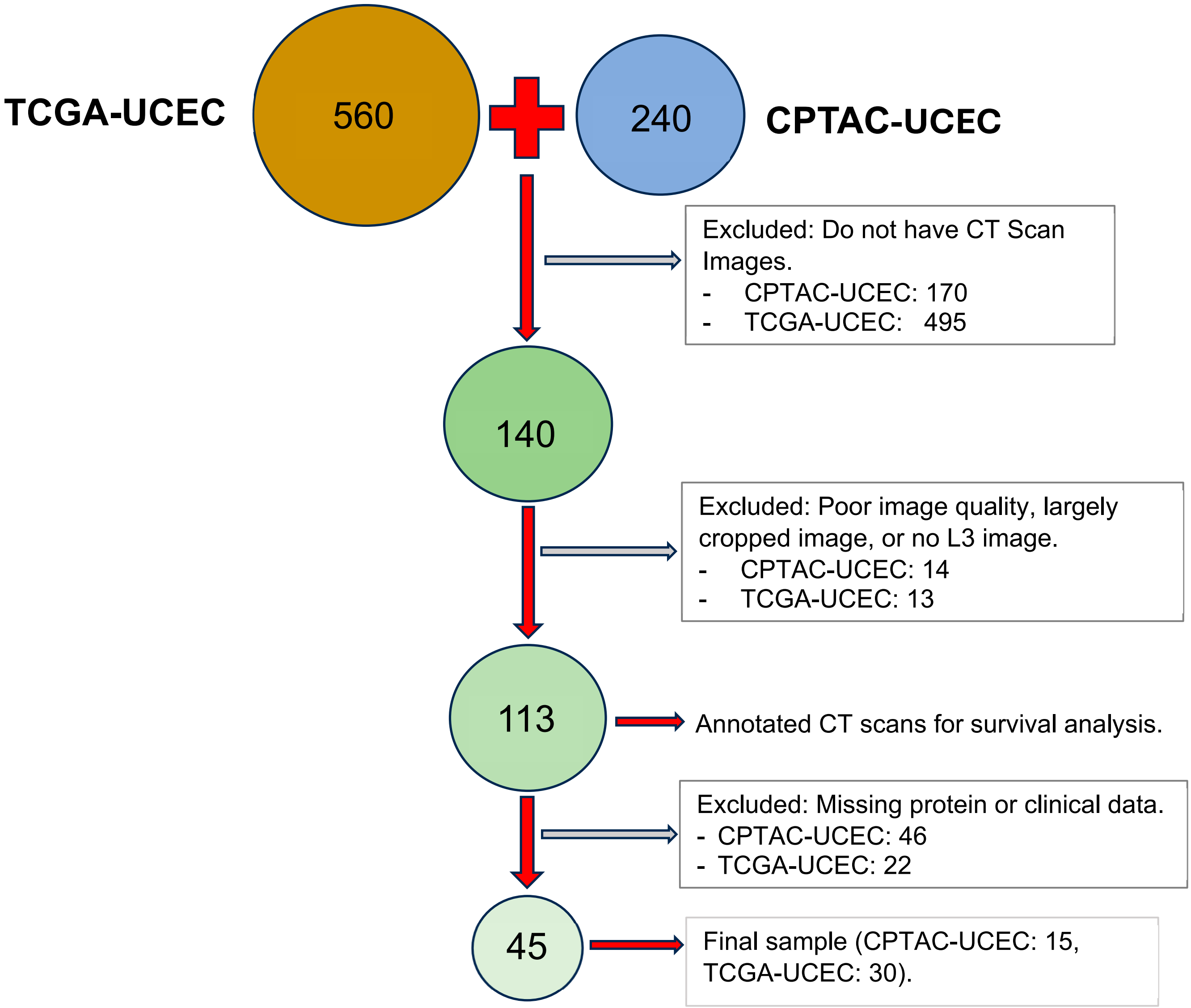

Supplemental Figure S2: Patient selection flow chart.

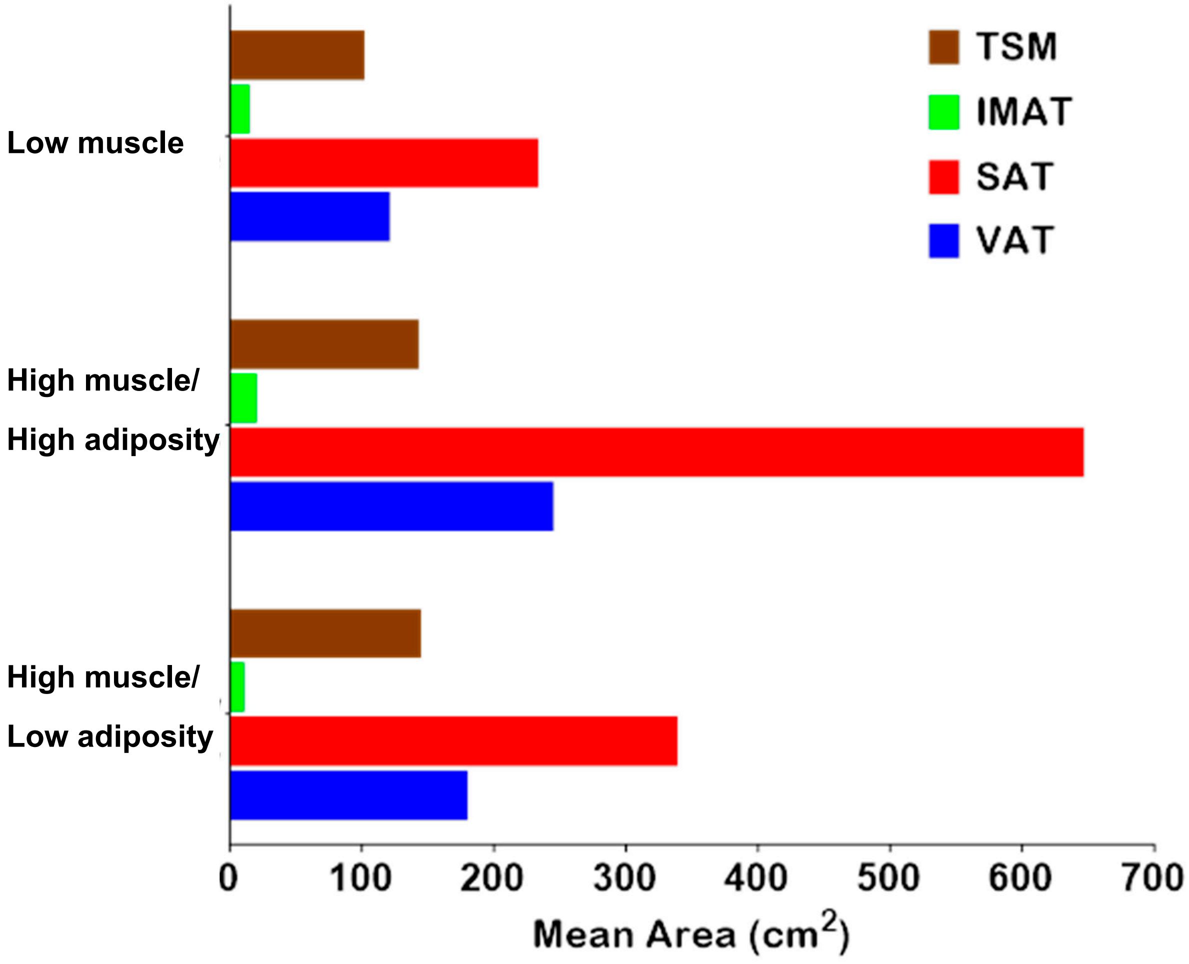

Supplemental Figure S3: Total muscle and adipose tissue distribution within the body composition types.

Total skeletal muscle (TSM), intramuscular adipose tissue (IMAT), subcutaneous adipose tissue (SAT), and visceral adipose tissue (VAT) surface areas were measured from the transverse (horizontal) plane CT scan at the L3 vertebrae level. The mean surface area for every tissue in the body composition categories was computed and is presented in the graph above.

A

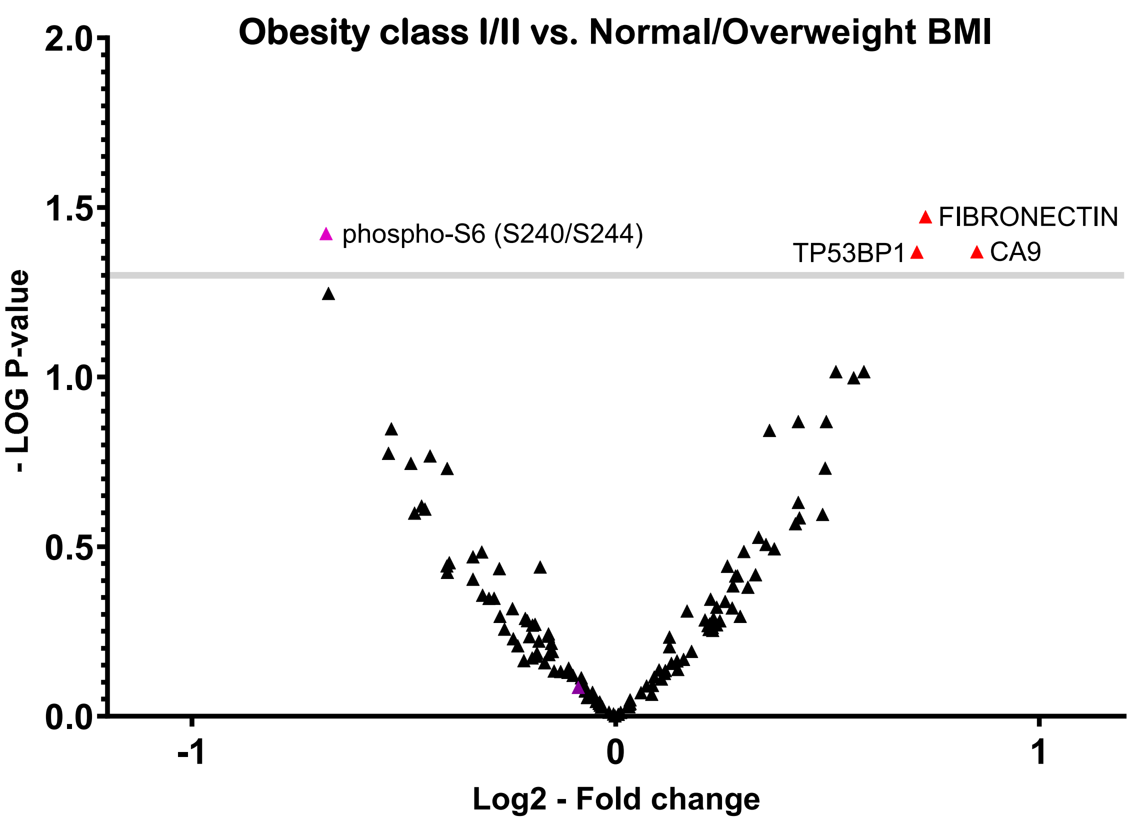

Obesity class I/II vs. Normal/Overweight BMI
- LOG P-value
2.0
1.5
1.0
0.5
0.0
-1
0
1
Log2 - Fold change
phospho-S6 (S240/S244)
FIBRONECTIN
TP53BP1
CA9

B

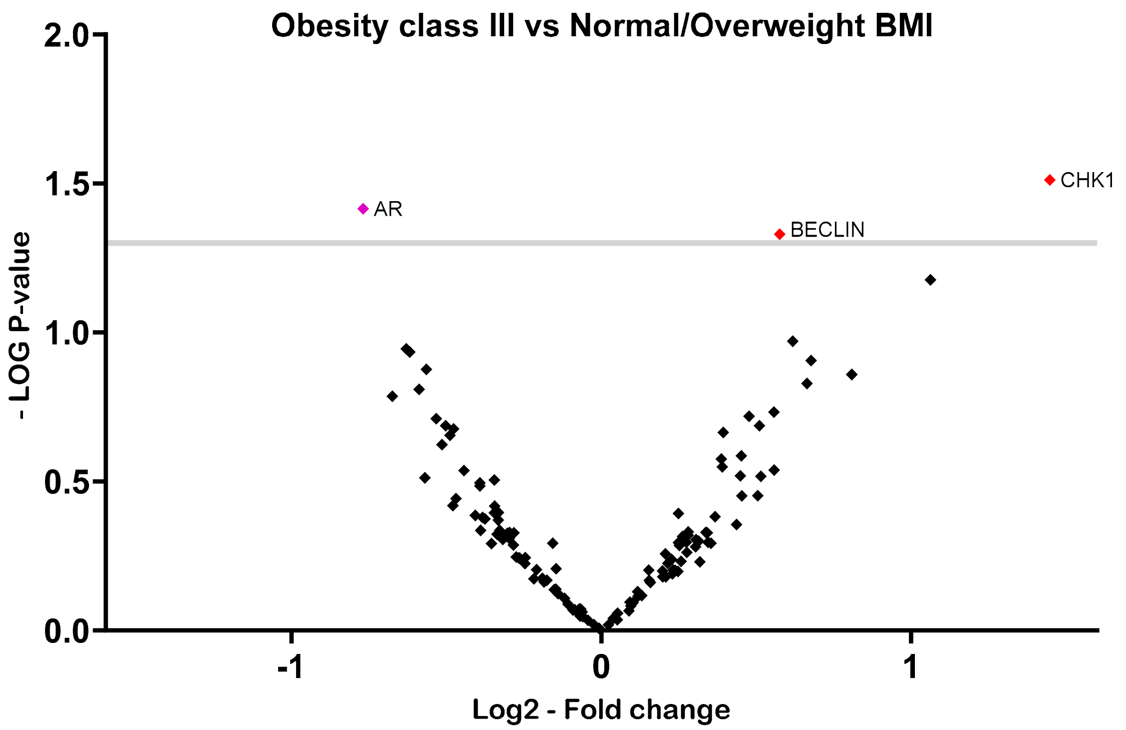

Supplemental Figure S4. Volcano plot of differential expression of protein tumors based on BMI categories with normal/overweight as a referent group.

Supplemental File S1: Data sheets in the Excel document

1: Complete list of proteins from TCGA and matches from CPTAC.

2: Regression analysis results for the association between body composition (High muscle/Low adiposity as a reference group) and tumor protein expressions.

3: Regression analysis results for the association between the BMI (normal/overweight BMI group as a reference group) and tumor protein expressions.
